# Automated MoCA scoring for Arabic speakers using hybrid AI of multimodal speech, vision, and LLM integration

**DOI:** 10.3389/fpsyg.2026.1833118

**Published:** 2026-07-08

**Authors:** Yara Jehad Rabaya, Sherin Asad Qarariya, Tuqa Murad Abualhaija, Asem A. Salah, Huthaifa I. Ashqar

**Affiliations:** Computer Systems Engineering Department, Arab American University, Jenin, Palestine

**Keywords:** Arabic NLP, artificial intelligence, cognitive impairment, dementia screening, explainable AI, MoCA, multimodal, neuropsychological assessment

## Abstract

**Introduction:**

Early detection of dementia and mild cognitive impairment (MCI) remains a significant clinical challenge, particularly in Arabic and resource-limited settings where culturally adapted screening tools are scarce and access to specialized neuropsychological services is constrained. The Montreal Cognitive Assessment (MoCA) is a widely validated instrument for detecting MCI; however, its administration is largely manual, limiting scalability for large-scale community screening.

**Methods:**

In this study, we present a preliminary feasibility study of an AI-powered hybrid multimodal cognitive screening system that digitizes the Arabic version of the MoCA and integrates speech processing, computer vision, and large language model-based reasoning within a unified platform. The system captures verbal and visuospatial responses via smartphone sensors, extracts structured linguistic and geometric features, and performs cognitive state classification using a Qwen-based structured reasoning framework with emphasis on transparency and interpretability. The system was evaluated using a custom Arabic dataset of 24 participants from an elderly care facility in Palestine, including cognitively normal individuals, those with MCI, and dementia cases. This pilot evaluation is intended to establish initial feasibility rather than definitive clinical equivalence, and the findings require validation through larger, adequately powered multisite investigations.

**Results and Discussion:**

The proposed hybrid approach achieved an overall diagnostic agreement of 83.3% with manual clinical scoring, a Cohen’s *κ* of 0.74 (substantial agreement), and demonstrated complete output stability across five independent runs. No severe cross-category misclassifications (i.e., from Dementia to Normal) were observed. After excluding two technically compromised audio recordings, adjusted accuracy reached 90.9% with *κ* = 0.86. These findings support the feasibility of developing interpretable, culturally adapted AI tools for scalable cognitive screening among Arabic-speaking populations in low-resource environments. The proposed system is intended as an assistive first-line screening tool and is not a replacement for professional medical evaluation.

## 1 Introduction

Dementia is a progressive neurodegenerative disorder characterized by deterioration in memory, executive functions, language, and visuospatial abilities. Early detection is critical, as a prolonged preclinical phase may extend for 10–20 years before overt cognitive symptoms emerge, defining a critical window for early intervention (Li et al., 2022). Mild Cognitive Impairment (MCI) represents a transitional stage within this trajectory, offering an opportunity for monitoring before progression to more advanced stages (Cavedoni et al., 2020).

However, early symptoms are frequently subtle and may be misattributed to normal aging, particularly in Arabic-speaking communities where culturally adapted cognitive screening tools remain limited (Kabalan et al., 2025; Taiebine, 2024). A major challenge is the limited availability of scalable, culturally and linguistically adapted diagnostic instruments. Although standardized assessments such as the Montreal Cognitive Assessment (MoCA) have been translated into Arabic and validated for detecting MCI (Rahman and El Gaafary, 2009), their administration remains largely manual and dependent on trained specialists.

Traditional pen-and-paper tests are time-consuming and insufficiently optimized for detecting preclinical decline, leading to increasing interest in AI-assisted cognitive screening (Li et al., 2022). While AI-based systems have shown promising diagnostic performance, questions remain regarding their cultural adaptability, interpretability, and validation across diverse healthcare contexts (Li et al., 2022; Kabalan et al., 2025). Dialectal variation, bilingualism, and educational disparities further influence cognitive test performance in Arabic-speaking populations (Taiebine, 2024).

Conventional clinical diagnosis frequently relies on the manifestation of significant memory loss or behavioral symptoms rather than systematic early screening (Rahman and El Gaafary, 2009). This delay underscores the need for scalable, culturally aligned, and clinically reliable digital screening tools that are transparent and explainable.

As a result, this study makes the following primary contributions to the field of AI-assisted cognitive screening:

- A hybrid explainable AI framework that digitizes the full Arabic MoCA (Version 8.1), combining deterministic rule-based scoring for objective tasks with constrained Large Language Model (LLM)-based reasoning (Qwen2.5-7B-Instruct) for semantically complex domains.
- The first publicly released Arabic MoCA multimodal dataset (audio recordings, visuospatial drawings, and touch interaction logs) from a real-world clinical cohort in Palestine, available at https://github.com/yarajehadrabaya/arabic-moca-ai-dataset.
- A three-tier safety architecture incorporating a deterministic override mechanism that prevents underestimation from LLM variability, achieving zero severe cross-category misclassification errors (i.e., from Dementia to Normal).
- Empirical validation demonstrating 83.3% overall diagnostic agreement (Cohen’s *κ* = 0.74) with full manual MoCA scoring, rising to 90.9% (κ = 0.86) after excluding technically compromised audio recordings.
- Complete output reproducibility across five independent system runs, confirming operational stability of the hybrid pipeline despite inclusion of non-deterministic LLM components.

## 2 Literature review

### 2.1 The Montreal cognitive assessment: validation and limitations

The MoCA was introduced by Nasreddine et al. (2005) as a 10-min, 30-point cognitive screening instrument designed specifically to detect MCI in patients who score within the normal range on the Mini-Mental State Examination (MMSE). The original validation study demonstrated 90% sensitivity for MCI and 100% sensitivity for mild Alzheimer’s disease with a cutoff of 26, substantially outperforming the MMSE’s 18% MCI sensitivity. Since its publication, the MoCA has been referenced in over 26,000 scientific articles and translated into more than 100 languages and dialects, establishing it as the gold-standard brief cognitive screening instrument in both clinical and research settings.

A subsequent meta-analysis by Malek-Ahmadi and Nikkhahmanesh (2024) synthesized 55 observational studies comprising 17,343 cognitively unimpaired and 8,413 amnestic MCI subjects, reporting pooled AUC values ranging from 0.71 to 0.99 across studies. The analysis identified optimal MoCA cutoff variability across populations and recommended a cutoff of 24 for detecting suspected impairment, which reflected the well-documented influence of age, education, and cultural background on MoCA performance. These demographic sensitivities are particularly pronounced in Arabic-speaking populations, where educational disparities, dialectal variation, and limited normative data compound the challenge of culturally valid screening (Kabalan et al., 2025; Taiebine, 2024).

Despite the MoCA’s clinical value, its reliance on trained administrator supervision significantly limits scalability in low-resource settings. The need for specialist involvement, standardized paper materials, and expert scoring constrains population-level deployment, motivating the development of automated and digitized alternatives (Rahman and El Gaafary, 2009). Digital self-administered tools such as MoCA XpressO (Klil-Drori et al., 2024) have begun exploring this space, though culturally specific adaptations for Arabic-speaking populations remain extremely limited.

### 2.2 Computerized and AI-assisted cognitive assessment

A systematic review of computerized cognitive assessment tools for MCI and dementia by Henkel et al. (2025) identified 26 studies meeting inclusion criteria, reporting sample sizes ranging from 22 to 4,486 participants and convergent validity correlations with standard tests of *r* = 0.30 to 0.90. Critically, the review found that the most diagnostically effective tools integrated multiple cognitive domains, including spatial working memory, delayed recall, and semantic fluency, rather than single-domain approaches. Machine learning applied to the digital Clock Drawing Test (dCDT) achieved 80%–90% accuracy distinguishing MCI from Alzheimer’s dementia, which highlighted visuospatial features as particularly information-rich biomarkers.

Complementary evidence from machine learning analyses of MoCA domain scores in Parkinson’s disease (Jeon et al., 2022) demonstrated that item-level Machine Learning (ML) approaches including logistic regression, support vector machines, and random forest achieved accuracy of 0.87–0.89 when combined with cognitive complaint variables, substantially outperforming conventional cutoff-based classification of 0.66. This finding emphasizes that task-level score decomposition, rather than aggregate score thresholding, extracts substantially more diagnostic information from cognitive assessments. The present study applies a directly analogous domain decomposition strategy to the Arabic MoCA.

A further systematic review by Gourdeau et al., 2025 demonstrated that machine learning models integrating MoCA subtest scores, demographic variables, and cognitive chart metrics consistently outperformed single global-threshold approaches across diagnostic categories. The study validates the use of SHAP values and cognitive chart-derived metrics including Cognitive Quotient, standardized age as interpretable, clinically meaningful contributors to AI-based MoCA scoring, which is a perspective aligned with our hybrid explainability design.

An important distinction must be drawn between the present study’s approach and a parallel line of work in which multimodal features are fed directly into a classifier trained to predict cognitive status, with the MoCA score used only as a ground-truth label. Several international groups have pursued this paradigm using instrumented pen and tablet devices: for instance, McMurray et al. (2024) utilized a Dot-matrix digital pen to capture real-time handwriting kinematics and combined these with concurrent speech features to classify MCI from healthy controls, achieving high diagnostic accuracy without requiring explicit task scoring. Similarly, Kourtis et al. (2019) demonstrated that passive digital biomarkers extracted from a tablet-administered cognitive battery could discriminate early Alzheimer’s disease with AUC > 0.85. In these approaches, the MoCA score, or a derived clinical label, serves purely as a training target; the system learns an implicit mapping from raw multimodal features to diagnostic category. While this paradigm can achieve strong discriminative performance, it produces a black-box classifier with limited task-level interpretability and requires substantial labeled training data. By contrast, the present study implements automated scoring of each MoCA sub-task independently, mirroring the clinical administration procedure and preserving the full domain-level score breakdown that clinicians rely upon for interpretable assessment. This automated scoring approach is fundamentally different: rather than predicting cognitive status from features, the system replicates the structured scoring logic of the MoCA instrument itself, making the output directly comparable to a human-administered MoCA and inherently transparent at the task level. The hybrid architecture thus occupies a distinct niche between purely feature-based classifiers and fully manual administration, combining the scalability advantages of automation with the clinical interpretability of standardized neuropsychological scoring.

### 2.3 Speech and natural language processing for dementia detection

Natural language processing (NLP) of verbal output has emerged as one of the most studied non-invasive biomarker modalities for cognitive decline detection. A systematic review by Shankar et al. (2025a,b) analyzed 51 NLP studies across 8 databases involving 17,340 participants with mean age of 72.4 years, finding that combined linguistic and acoustic approaches achieved the highest diagnostic accuracy (mean 87%; AUC 0.89). Critically, this outperformed either linguistic-only (mean 83%; AUC 0.85) or acoustic-only (mean 80%; AUC 0.82) approaches. Lexical diversity, syntactic complexity, and semantic coherence were the most consistently predictive features across cognitive conditions, which are markers that are directly targeted by MoCA language tasks.

The foundational work of Petti et al. (2020) systematically reviewed 72 deep learning studies for dementia diagnosis from speech, establishing that ASR-based pipelines introduce significant variability depending on dialectal coverage and recording quality. These ASR sensitivity issues are especially critical for dementia data where important cognitive indicators such as repetitions and silent pauses can be lost during transcription, which is a challenge that is compounded for Arabic, whose dialectal diversity substantially exceeds that of well-studied languages like English and Mandarin. Our study directly addresses this by implementing Arabic-specific normalization and error correction pipelines prior to feature extraction.

A comprehensive scoping review of NLP for dementia by Peled-Cohen and Reichart (2025) further highlighted a critical gap: while English-language corpora (notably the DementiaBank Pitt Corpus) are well studied, approximately 60% of global dementia patients are non-English speakers from low and middle-income countries, yet these populations are severely under-represented in NLP research. The review emphasized that translation-based transfer learning shows promise but requires extensive validation due to grammatical complexity and dementia-specific nuances across languages. This gap directly motivates the Arabic-specific clinical dataset and NLP pipeline developed in the present study.

Speech-based prediction of Alzheimer’s progression was further demonstrated in the landmark study by Amini et al. (2024) in Alzheimer’s & Dementia, which applied NLP to audio recordings from the Framingham Heart Study to predict MCI-to-AD conversion within 6 years, emphasizing that linguistic features alone, without acoustic or imaging data, provide clinically actionable predictive signal. This establishes the validity of text-based reasoning from verbal responses as a legitimate cognitive screening strategy, which supports our system’s ASR-to-LLM scoring approach for verbal MoCA tasks.

### 2.4 Large language models in cognitive assessment and clinical NLP

The rapid advancement of LLMs has introduced qualitatively new capabilities for clinical language understanding (Ashqar R. I. et al., 2025a,b). Harrison et al. (2025) provided a systematic review of LLMs in dementia care and research, noting that speech analysis for cognitive impairment detection represents the most mature clinical application of LLMs, with GPT-3 and GPT-4-based approaches demonstrating promising performance in distinguishing Alzheimer’s patients from healthy controls through analysis of language patterns, word-finding difficulty, and semantic coherence. The review identified structured prompting and hybrid (i.e., LLM and rule-based) architectures as priority directions for maintaining clinical reliability.

Du et al. (2024) at Mass General Brigham conducted a comparative study of LLMs for detecting early cognitive decline from electronic health record (EHR) clinical notes, evaluating GPT-4 and Llama 2 against traditional ML models on 4 years of longitudinal EHR data. The study found that LLMs and traditional models exhibit complementary error profiles, and that ensemble approaches combining LLMs with local clinical knowledge substantially improved diagnostic performance, which supports the hybrid architecture design of the present study, which explicitly separates deterministic algorithmic components from LLM-mediated semantic reasoning.

Kashyap et al. (2025) proposed an LLM-aided feature engineering approach that extracts clinically relevant features from the Oxford Textbook of Medicine using GPT-4 for dementia subtype classification. Their work underscores that LLM-derived representations can complement traditional neuropsychological tools while preserving clinical interpretability through structured feature design, which is a principle central to the Qwen2.5-7B prompting strategy in our system. Critically, the study also identifies LLM hallucination and inconsistency as persistent risks in high-stakes clinical settings, reinforcing the importance of deterministic safety override mechanisms such as those implemented in our hybrid memory module.

For the specific challenge of Arabic language processing in clinical contexts, a scoping review of Arabic NLP for mental health by Alasmari (2025) documented the evolution from traditional ML including RNN and lexicons to transformer-based models (AraBERT, CAMeL) from 2022 onward. The review confirmed that Arabic NLP performance lags substantially behind English due to data scarcity, dialectal diversity, and limited domain-specific pre-training. These constraints directly motivate our decision to deploy a general-purpose multilingual Qwen2.5-7B model under structured Arabic-specific prompting constraints rather than relying on dialect-specific fine-tuned models that may lack the clinical reasoning capacity needed for MoCA semantic evaluation.

### 2.5 Explainability and clinical safety in AI-assisted cognitive screening

The imperative for explainable AI in clinical cognitive screening has been progressively formalized. Tjoa and Guan (2020) provided a landmark survey of explainable artificial intelligence (XAI) methods for medical AI, distinguishing between post-hoc interpretability methods and intrinsically transparent architectures. The survey strongly advocates for hybrid systems in high-stakes clinical settings, arguing that black-box end-to-end classifiers, while often achieving higher aggregate accuracy, fail to provide the decision traceability required for clinical trust, regulatory approval, and professional accountability. These arguments are directly operationalized in our hybrid scoring framework, which maintains domain-level scoring traceability and restricts LLM involvement to semantically complex domains where deterministic rules are insufficient (Ashqar R. I. et al., 2025a; Ashqar H. I. et al., 2025b; Radwan et al., 2024).

A systematic review of explainable AI methods for speech-based cognitive decline detection (Shankar et al., 2025a,b) identified 13 studies between 2021 and 2025 from diverse geographic regions, finding that XAI-enhanced systems consistently achieved performance comparable to black-box approaches while enabling the identification of clinically meaningful linguistic signatures. The review called for more diverse geographic and linguistic coverage, explicitly noting that Arabic-speaking populations are absent from the existing XAI cognitive screening literature, which is a gap our study directly addresses.

In the domain of visuospatial AI-based assessment, the clock drawing test has received particular attention. Deep learning classifiers for the dCDT have demonstrated AUC of 0.81–0.88 in detecting MCI and mild Alzheimer’s disease (Henkel et al., 2025; Klil-Drori et al., 2024). The validity and cultural generalizability of AI-based cognitive assessment was directly examined by Kalafatis et al. (2021) across 230 participants from multiple geographic cohorts of 95 healthy, 80 MCI, and 55 mild AD. The developed model achieved AUC of 81% for MCI and 88% for mild AD, with notably lower education bias than the MoCA (*r* = 0.17 vs. 0.34). This cross-cultural validation framework provides important benchmarking context for our study’s pilot cohort results, and the authors’ observation that AI model performance improved with increased training data underscores the importance of expanding our Arabic-specific dataset in future work.

### 2.6 Summary and research gap

Collectively, the literature demonstrates three converging trends: (1) digitized and AI-assisted cognitive assessment tools consistently achieve clinically meaningful agreement with manual MoCA or MMSE scoring when combining multimodal inputs with domain-specific feature extraction; (2) NLP and LLM-based verbal analysis provides strong complementary signal for cognitive classification, particularly for tasks requiring semantic understanding; and (3) explainable, hybrid architectures that preserve clinical scoring logic are increasingly recognized as the appropriate design paradigm for AI tools intended for clinical deployment.

Critically, to the best of our knowledge, no prior study has: (a) developed and clinically validated a hybrid AI-assisted MoCA scoring system specifically for Arabic-speaking populations; (b) released a multimodal Arabic cognitive assessment dataset from a real-world clinical cohort; or (c) demonstrated the use of a constrained LLM (Qwen2.5-7B-Instruct) under Arabic-specific clinical prompting constraints for MoCA task scoring with deterministic safety overrides. The present study addresses all three gaps simultaneously, which represents a direct contribution to the intersection of culturally adapted cognitive AI, Arabic clinical NLP, and explainable medical decision support.

## 3 Dataset

### 3.1 Overview

**Figure 1:**
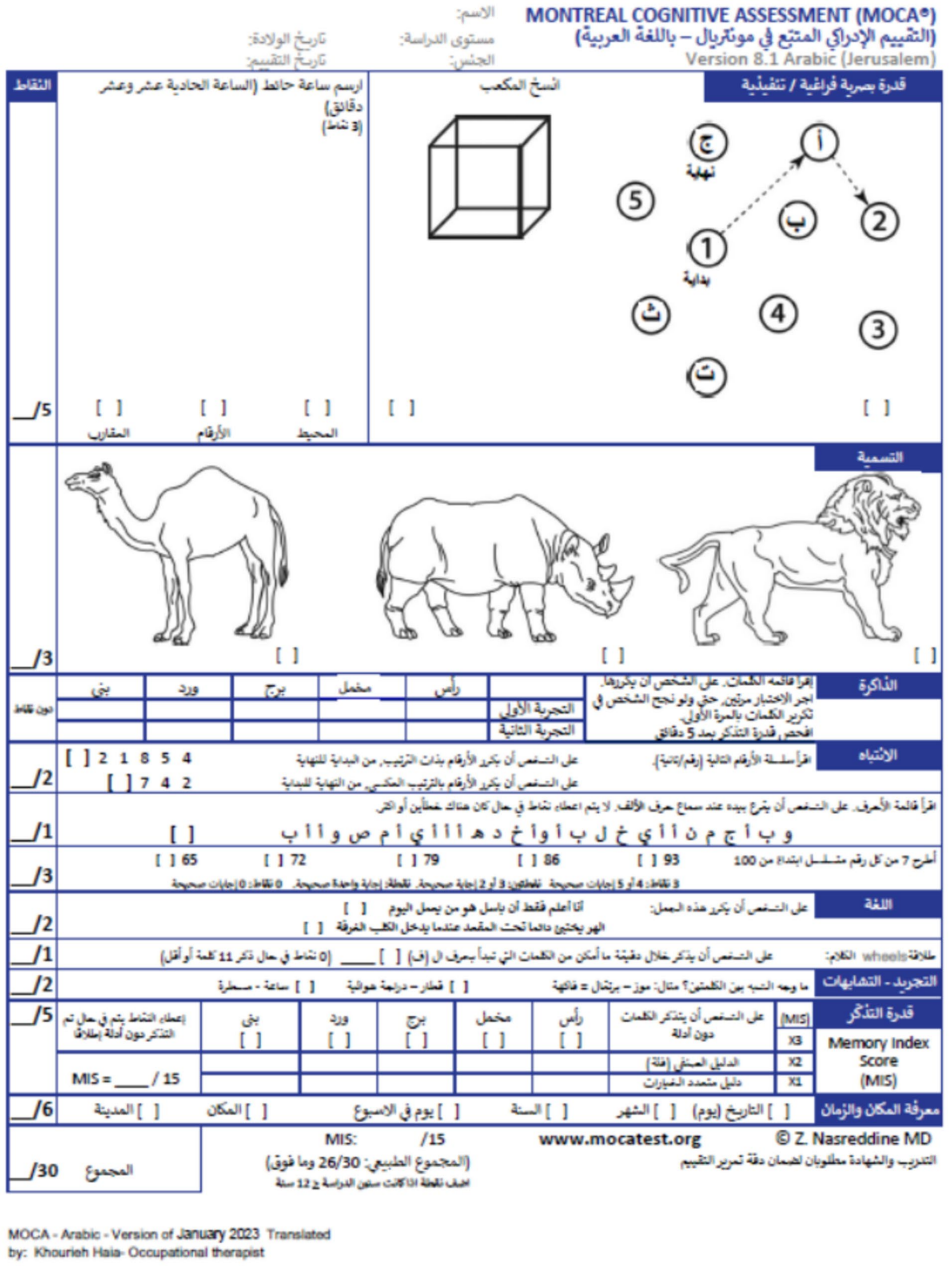
Official MoCA Version 8.1 Arabic form.

The Arabic MoCA AI Dataset was collected specifically for this study and is publicly available at https://github.com/yarajehadrabaya/arabic-moca-ai-dataset. It is the first multimodal dataset for Arabic cognitive screening derived from a real-world clinical cohort, and it supports reproducibility of the experimental results reported herein. Within the structure of the smart application developed for this study for early detection of dementia, the Arabic version of the MoCA has been adopted as a tool for assessing the cognitive functions of users in the process of early detection of dementia, which is shown in Figure 1. We digitized the form and integrated it into the application so that it allows users to perform the required tasks such as drawing a clock or repeating words, for the system to analyze them automatically using computer vision and natural language processing techniques.

### 3.2 Participants

**Figure 2:**
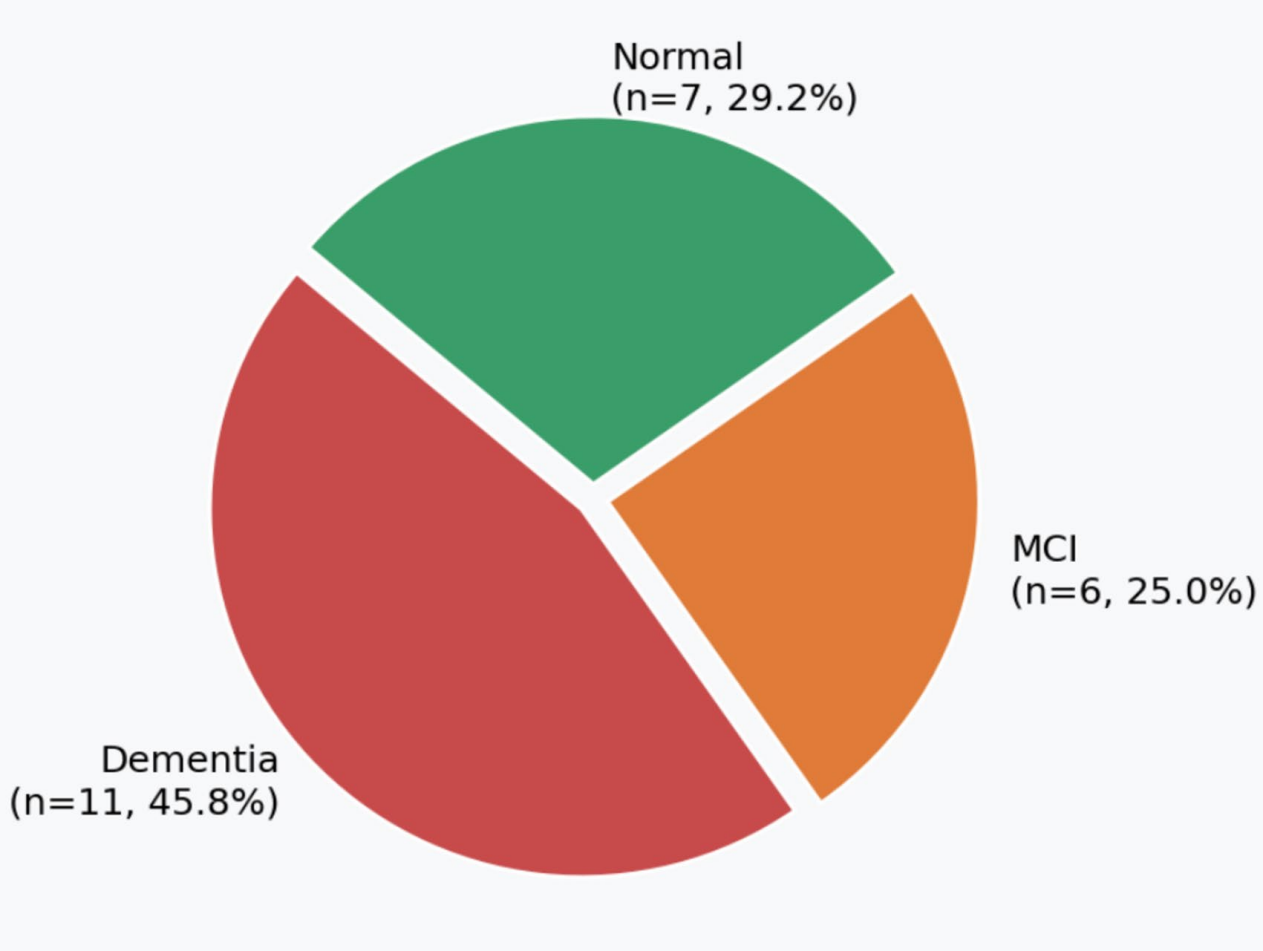
Participant distribution by cognitive status (*N* = 24). Dementia cases constituted the largest group (45.8%), followed by cognitively normal (29.2%) and MCI (25.0%) participants.

A total of *N* = 24 Arabic-speaking adults aged 50 years and older were recruited prospectively from an elderly care facility in Palestine with the cooperation of the facility administration and supervising clinical staff. Participants were classified into three clinically documented cognitive categories based on prior medical evaluation and manual MoCA assessment under official Version 8.1 thresholds (Normal: S ≥ 26; MCI: 18 ≤ S ≤ 25; Dementia: S ≤ 17), as shown in Figure 2. The cohort comprised 14 female and 10 male participants (58.3% female, 41.7% male). Educational attainment ranged from no formal schooling to secondary education, with 10 participants (41.7%) classified as having low educational attainment (0–6 years), 9 (37.5%) as moderate (7–12 years), and 5 (20.8%) as higher secondary or equivalent. As specified in the official MoCA Version 8.1 administration guidelines, one correction point was added to the raw MoCA score for participants with 12 years or fewer of formal education, and this adjustment was applied consistently to both manual and AI-derived total scores. A point-biserial correlation between educational attainment (years) and total manual MoCA score yielded *r* = 0.31 (*p* = 0.14), indicating a moderate positive trend consistent with the well-documented education effect on MoCA performance, though not reaching statistical significance in this small sample. Regarding gender, the mean manual MoCA score was 16.8 (SD = 7.1) for female participants and 18.4 (SD = 8.3) for male participants; a Mann–Whitney U test revealed no statistically significant difference (*U* = 58, *p* = 0.54), consistent with the absence of a strong gender effect on MoCA total scores reported in the normative literature for this age group. These analyses are exploratory given the sample size and are reported for transparency; adequately powered subgroup analyses will be conducted in the planned full-scale validation study.

Participants were included if they were aged 50 years or older, were native or fluent Arabic speakers, were residents of the care facility at the time of data collection, and had a prior clinical cognitive evaluation on record. The following exclusion criteria were applied: (1) uncorrected visual impairment that would prevent completion of visuospatial tasks (e.g., clock drawing, cube copy, trail making), as confirmed by care facility nursing records; (2) functional illiteracy or inability to comprehend verbal instructions in Arabic, which would preclude valid administration of language-dependent tasks; (3) primary neurological speech or language disorders including dysarthria, aphasia, or significant motor speech impairment that would confound AI-based audio analysis independent of cognitive status; (4) active clinical depression or other major psychiatric comorbidity documented in the facility medical record at the time of assessment, given the well-established influence of depressive symptomatology on MoCA performance; and (5) inability or unwillingness to provide informed assent for data collection. These criteria were assessed by the supervising clinical psychologist and nursing staff prior to enrollment. No participant was excluded solely on the basis of cognitive severity.

### 3.3 Multimodal data modalities

Each assessment session produced three distinct data modalities:

- *Audio recordings*: Verbal responses to MoCA tasks including naming, attention, language, and memory domains, captured via smartphone or external USB microphones. Files were stored as individual per-task recordings to facilitate independent processing.
- *Visuospatial drawing images*: Photographs of hand-drawn clock and cube copy tasks completed on paper under standardized lighting conditions, processed through an OpenCV computer vision pipeline.
- *Touch interaction logs*: Timestamped JSON logs of finger trajectories from the digital Trail Making Test (TMT), including absolute and normalized screen coordinates and task completion timing.

### 3.4 Data collection procedure

Data were collected during supervised in-person assessment sessions at an elderly care facility. Sessions were conducted in quiet rooms and lasted approximately 15–20 min each. The assessment was delivered via a custom mobile application (Android/web-based) replicating the standardized MoCA procedure, supervised by two nursing staff and a clinical psychologist. Instructions were delivered verbatim according to official MoCA Version 8.1 Arabic guidelines. All data were anonymized using coded participant identifiers (P001-P024 range). Regarding the test–retest design and the potential for practice effects: it is important to note that the AI-based system did not constitute a second administration of the MoCA that participants completed independently of the manual assessment. Rather, the two evaluation methods used the same single administration session. Each participant completed the MoCA tasks once, with responses captured simultaneously by both the manual scoring clinician and the AI system’s multimodal recording apparatus (audio recordings, drawing photographs, and digital touch logs). The manual MoCA score was computed by the supervising clinical psychologist in real time during the session, while the AI system processed the stored multimodal data independently and offline after the session concluded. There was therefore no repeat exposure to the MoCA items, no separate AI-administered session, and no risk of practice effects or carryover learning between administrations. The manual score served as the reference standard, and the AI score was computed *post hoc* from the same session’s recorded data. This single-session concurrent design ensures that any agreement or disagreement between the two scoring methods reflects differences in evaluation logic rather than task familiarity effects.

### 3.5 Ethics

The study was approved by the Arab American University Institutional Review Board in Ramallah under the code number of R-2026/A/98/N. This study was conducted as a non-interventional observational validation study in accordance with the ethical principles of the Declaration of Helsinki and did not involve any therapeutic intervention or modification of standard clinical care. All participants, or their legally authorized representatives where applicable, provided written informed consent prior to enrollment, following a full verbal and written explanation of the study purpose, voluntary nature of participation, right to withdraw at any time without consequence, and data handling procedures. All collected data were fully anonymized prior to analysis using coded participant identifiers (e.g., P001-P024); no personally identifiable information was retained in the analytical dataset. Audio recordings, drawing images, and interaction logs were stored and processed exclusively under these anonymized identifiers. The AI system functioned strictly as an evaluative research tool and did not influence clinical decision-making or alter the standard care provided to any participant.

## 4 Materials and methods

### 4.1 Study design

This study was designed as a prospective observational preliminary feasibility study evaluating the diagnostic agreement between a hybrid AI-assisted MoCA scoring framework and standardized manual MoCA assessment. By design, a pilot feasibility study of this nature is not intended to yield definitive or generalizable conclusions about clinical equivalence; rather, its purpose is to establish proof of concept, identify system failure modes, and generate the performance estimates needed to power a subsequent full-scale validation trial. It is important to define the precise nature of this comparison. The AI system is not a trained or fine-tuned machine learning classifier; it is an orchestrated pipeline of pre-existing components, specifically OpenCV-based geometric algorithms, an open-source Arabic ASR model, and the pre-trained Qwen2.5-7B-Instruct LLM, integrated under structured scoring rules that replicate official MoCA Version 8.1 administration criteria. The reference standard is manual MoCA scoring completed by trained clinical staff in accordance with official MoCA Version 8.1 guidelines, prior to and independent of AI-based evaluation. The comparison is therefore between algorithmic heuristics implementing standardized psychometric criteria and human-administered psychometric scoring according to those same criteria. The study does not compare the system against specialist neurological diagnosis, imaging-based biomarkers, or DSM-5 diagnostic criteria. This scope is appropriate for a first-line screening tool feasibility study and is consistent with the intended application of the system as an automated analog of the paper-based MoCA administration. A formal *a priori* sample size calculation for a definitive equivalence study, targeting 80% power at *α* = 0.05 to detect a clinically meaningful difference in kappa of Δ*κ* = 0.15, would require approximately 150–200 participants across three cognitive strata. The present pilot cohort of *n* = 24 was not selected to satisfy this requirement, but rather to assess feasibility within the constraints of the available clinical setting. The roadmap for follow-up investigation is outlined in Section 5.1. Manual scoring was completed prior to AI-based evaluation and served as the reference standard for diagnostic classification based on official MoCA cut-off thresholds.

Diagnostic performance was assessed by comparing AI-generated classifications against manual reference classifications across three cognitive categories. Agreement analysis included overall accuracy, class-specific sensitivity, confusion matrix evaluation, and Cohen’s kappa (κ) coefficient to quantify inter-method agreement beyond chance.

### 4.2 System architecture overview

**Figure 3:**
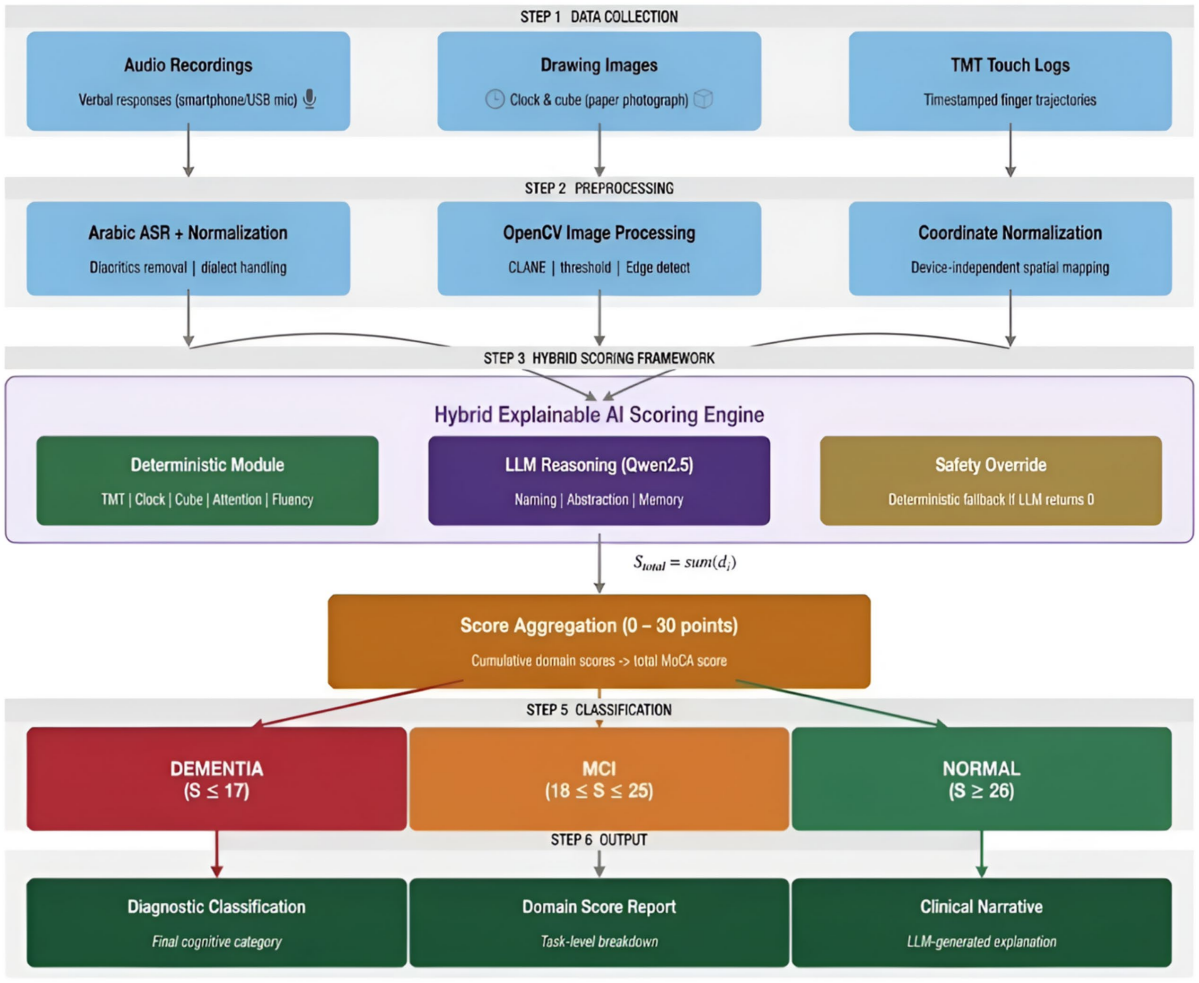
System processing pipeline of the Hybrid AI-Assisted MoCA scoring system. The framework integrates deterministic algorithmic modules, constrained LLM reasoning, and a safety override mechanism. Data flows from three input modalities through preprocessing and hybrid scoring to produce a final cognitive classification and interpretable clinical narrative.

The system architecture is illustrated in Figure 3 as a flowchart. The pipeline proceeds through six functional stages including multimodal data collection, domain-specific preprocessing, hybrid scoring, score aggregation, clinical classification, and structured output generation.

### 4.3 Hybrid explainable AI scoring framework

The proposed system implements a layered hybrid scoring architecture combining deterministic algorithmic validation with constrained LLM reasoning. The framework was explicitly designed to preserve clinical interpretability, scoring traceability, and adherence to official MoCA criteria while enabling multimodal automation. Unlike end-to-end black-box classifiers, the system separates four distinct computational layers including deterministic geometric and arithmetic validation, linguistically normalized symbolic evaluation, constrained LLM-mediated semantic reasoning, and rule-based safety override mechanisms.

The total MoCA score ( $S_{total}$ , 0 ≤ $S_{total}$ ≤ 30) is computed through cumulative domain aggregation of $S_{total}=\Sigma \;d_{i}$ , where $d_{i}$ represents the domain-level task scores derived using either deterministic algorithms or constrained LLM reasoning depending on task complexity.

#### 4.3.1 Deterministic computer vision modules (visuospatial domain)

Visuospatial tasks were evaluated using OpenCV-based geometric analysis. For the Clock Drawing task (0–3 points), the image was resized to 800 × 800 pixels and processed through CLAHE contrast enhancement, Gaussian smoothing, adaptive thresholding, and Canny edge detection. Three independent geometric validations were performed: (1) circle validity via contour circularity measure (4πA/P^2^ > 0.35); (2) numerical distribution via radial angular binning requiring at least 8 of 12 occupied zones; and (3) hand positioning via angular histogram peak detection. Each criterion contributed one point.

The circularity threshold of 0.35 was chosen empirically to accommodate the imprecise hand drawings typical of elderly participants while remaining discriminative against severely distorted or absent circles. To assess the robustness and sensitivity of this threshold, we retrospectively applied alternative threshold values of 0.25, 0.35, and 0.45 to all 24 clock drawing images. The threshold of 0.35 yielded the best agreement with manual clinical scoring (70.8%); a lower threshold of 0.25 introduced false-positive circle detections in three cases by accepting grossly non-circular shapes, while a higher threshold of 0.45 introduced false-negative rejections in two cases where clinicians judged the drawings as adequate. CLAHE contrast enhancement and adaptive thresholding were applied to mitigate the effects of variable lighting and paper background, and all data collection sessions were conducted under standardized indoor lighting conditions with white A4 paper and black ballpoint pens. The hard-coded geometric thresholds in the current pipeline represent a limitation in terms of open-environment generalizability. Factors including diverse pen stroke thickness, non-standard paper formats, camera angle variation, and outdoor or variable lighting conditions have not been systematically stress-tested. Integration of learned feature extraction layers or deep learning-based preprocessing modules to replace fixed geometric thresholds is identified as a priority improvement for future development.

For Cube Copy (0–1 point), evaluation used probabilistic Hough line transform and contour polygonal approximation. The Trail Making Test (0–1 point) validated stroke coordinates against predefined spatial targets using normalized Euclidean distance thresholds and sequential correctness matching.

#### 4.3.2 Deterministic attention and fluency modules

Speech responses underwent Arabic orthographic normalization, diacritic removal, and numeric extraction. Digit span tasks required exact sequence match. Serial subtraction was evaluated by verifying consecutive −7 transitions (0–3 points). For verbal fluency, words were tokenized, normalized, and deduplicated; a score of 1 was assigned for ≥11 unique valid words beginning with the target letter. Regarding Arabic dialectal variation: the participants in this study were native speakers of Palestinian Arabic (Levantine dialect), which differs substantially from Modern Standard Arabic (MSA/Fusḥā) in phonology, morphology, and lexical choice. Elderly participants in particular frequently produced responses in local colloquial registers, including non-MSA verb conjugations, regional vocabulary, and code-switching between dialect and MSA. The ASR component used was a multilingual Whisper-based model with Arabic support, which exhibits reduced word error rates on MSA compared to colloquial dialects. To mitigate this, a post-ASR normalization pipeline was applied: diacritic stripping, hamza normalization, ta’ marbuta standardization, and a custom Palestinian dialect lexicon mapping common colloquial forms to their MSA equivalents prior to feature extraction or LLM evaluation. For LLM-based scoring, prompts explicitly instructed Qwen2.5-7B to accept dialectal and colloquial Arabic variants as valid responses, to not penalize phonological simplification typical of elderly speech, and to evaluate semantic correctness rather than formal linguistic accuracy. We acknowledge that a formal linguistic evaluation quantifying ASR word error rates specifically on Palestinian elderly speech, including pathological speech patterns characteristic of dementia and MCI, was not conducted as part of this pilot study. Such an evaluation would require a speech corpus with manual transcription references, which does not currently exist for this population. Developing such a reference corpus and performing systematic ASR benchmarking across Palestinian and broader Levantine dialectal variation is identified as a key objective for subsequent validation work.

#### 4.3.3 LLM-constrained semantic evaluation modules

Tasks requiring semantic abstraction and flexible linguistic interpretation including Naming, Sentence Repetition, and Abstraction were evaluated using Qwen2.5-7B-Instruct under structured prompting constraints. Prompts instructed the model to accept colloquial Arabic, tolerate minor phonetic distortions, focus on shared conceptual meaning, and return output in fixed structured format (Score:[numeric], Analysis:[clinical explanation]). Scores were extracted programmatically via pattern matching. To ensure output determinism and suppress stochastic variation inherent to LLM inference, the model was configured with temperature = 0 and greedy decoding (do_sample = False, top_p = 1.0). This configuration effectively renders the LLM component deterministic given identical inputs, which is the mechanism underlying the complete score reproducibility observed across five independent runs (see Section 4.7).

It is important to clarify the nature of the LLM’s role in the explainability framework. The LLM does not function as a black-box end-to-end classifier. Rather, it serves as a constrained semantic evaluator for specific sub-tasks (Naming, Sentence Repetition, Abstraction) where deterministic rule-based scoring is insufficient due to the inherent flexibility of natural language responses. For each such task, the LLM is required to return a structured output consisting of a binary or integer score and a clinical explanation text that justifies the score assignment with reference to the participant’s actual verbal response. This structured output format means that every LLM-generated score is accompanied by a traceable, human-readable rationale, fulfilling the explainability requirement at the task level. The system does not derive any black-box aggregate classification from LLM outputs; all final classifications are computed from the cumulative sum of individually traceable domain scores using official MoCA thresholds. We acknowledge, however, that the LLM component does not currently extract formal neuropsychological feature metrics such as pause frequency, type-token ratio, or syntactic dependency depth. These quantitative linguistic biomarkers represent a recognized complementary signal for cognitive assessment (Shankar et al., 2025a,b), and their integration is identified as a priority direction for future development.

#### 4.3.4 Hybrid safeguarded memory module

Delayed recall implemented a three-layer hybrid mechanism: (1) deterministic phonetic matching with orthographic normalization and a strict fuzzy similarity threshold (SequenceMatcher ratio ≥ 0.85); (2) LLM interpretive reporting for structured clinical commentary; and (3) a safety override that adopts the deterministic matched count if the LLM returns zero despite confirmed matches. This safeguard prevented underestimation due to LLM variability.

The total hybrid score was categorized using official MoCA thresholds: Normal (26–30), MCI (18–25), and Dementia (0–17). No boundary optimization or machine-learned classification layer was applied, preserving strict comparability with manual clinical assessment.

## 5 Results

### 5.1 Participant distribution

A total of *N* = 24 Arabic-speaking participants completed the standardized Arabic MoCA assessment. Manual scoring served as the clinical reference standard. Based on official MoCA cut-off thresholds, the cohort comprised 11 participants with Dementia (45.8%), 6 with MCI (25.0%), and 7 cognitively Normal (29.2%) individuals.

### 5.2 Diagnostic agreement

**Figure 4:**
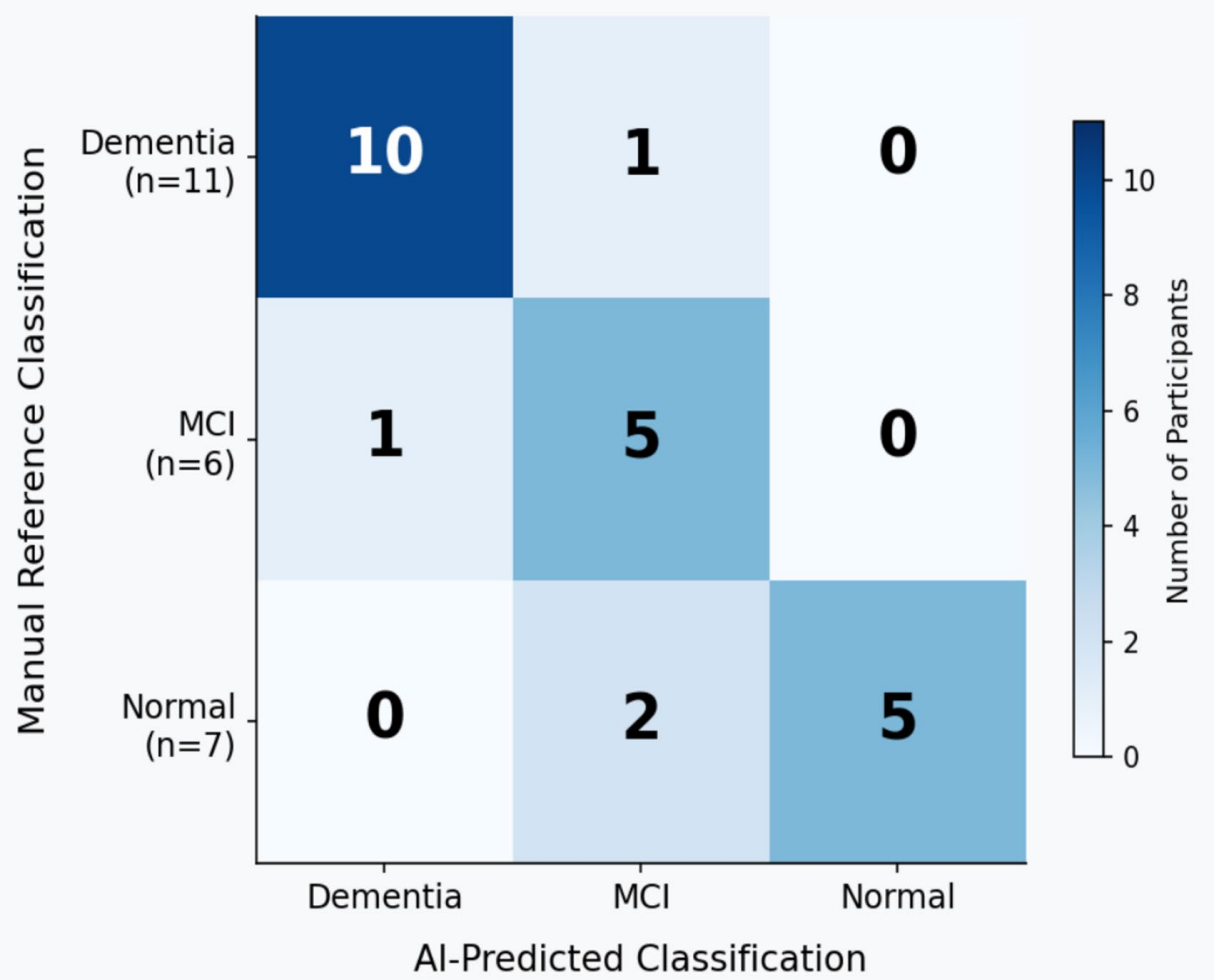
Confusion matrix of AI-predicted vs. manual reference MoCA classifications (*N* = 24). The heatmap shows correct classifications along the diagonal. No Dementia↔Normal transgressions occurred.

The confusion matrix comparing manual and hybrid AI-assisted classification is presented in Figure 4. A total of 20 out of 24 participants were correctly classified, yielding an overall diagnostic accuracy of 83.3%. No severe cross-category misclassification, specifically from Dementia to Normal, was observed. All four discrepancies occurred between adjacent categories including Dementia to MCI or MCI to Normal.

### 5.3 Overall performance metrics

**Figure 5:**
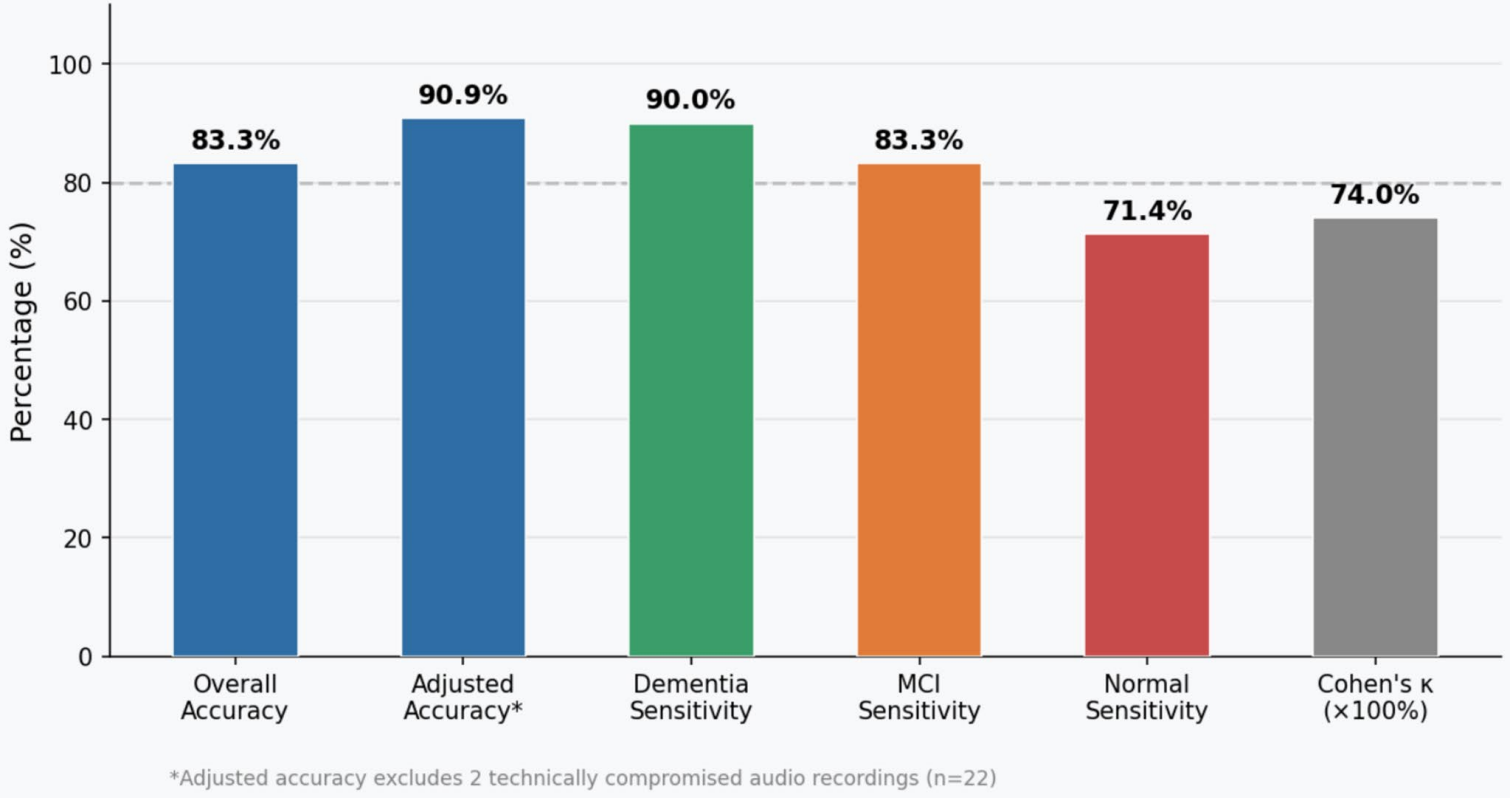
Summary of diagnostic performance metrics. Overall accuracy was 83.3%, rising to 90.9% after exclusion of two compromised recordings. Dementia sensitivity was highest (90.9%), while Normal sensitivity was lowest (71.4%). Cohen’s *κ* of 0.74 indicates substantial agreement.

Diagnostic performance metrics are summarized in Figure 5. The system achieved 83.3% overall accuracy (20/24), with class-specific sensitivities of 90.9% for Dementia, 83.3% for MCI, and 71.4% for Normal cognition. After excluding two technically compromised audio recordings (*n* = 22), adjusted accuracy reached 90.9%.

### 5.4 Agreement beyond chance (Cohen’s *κ*)

Observed agreement ( $P_{o}$ ) was 20/24 = 0.833. Cohen’s kappa coefficient was computed as *κ* = 0.74, corresponding to substantial agreement per Landis and Koch interpretation guidelines. After excluding the two technically compromised recordings, the adjusted observed agreement was 0.909 and the adjusted *κ* = 0.86, corresponding to almost perfect agreement.

### 5.5 Item-level agreement analysis

**Figure 6:**
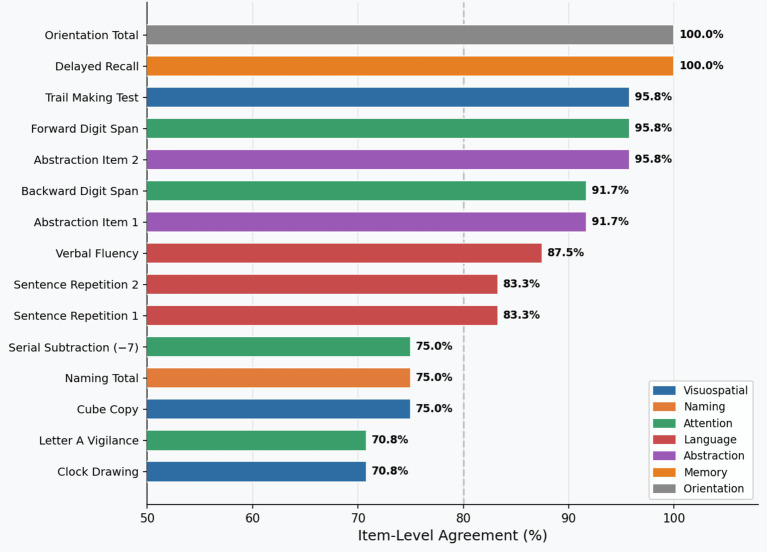
Item-level agreement between AI and manual MoCA scoring across all 15 sub-tasks (*N* = 24). Orientation and delayed recall achieved perfect agreement (100%). Tasks involving visual hand-drawn analysis (Clock Drawing) and auditory vigilance (Letter A) showed the lowest agreement (70.8%).

Item-level agreement between AI and manual scoring was analyzed across all MoCA sub-tasks. Orientation Total and Delayed Recall achieved perfect agreement of 100%. Trail Making Test and Forward Digit Span also reached near-perfect agreement of 95.8%. Clock Drawing and Letter a Vigilance showed the lowest agreement of 70.8% each, which reflects the inherent challenge of hand-drawn geometric interpretation and fine-grained auditory vigilance. Full results are presented in Figure 6.

### 5.6 Score distribution analysis

**Figure 7:**
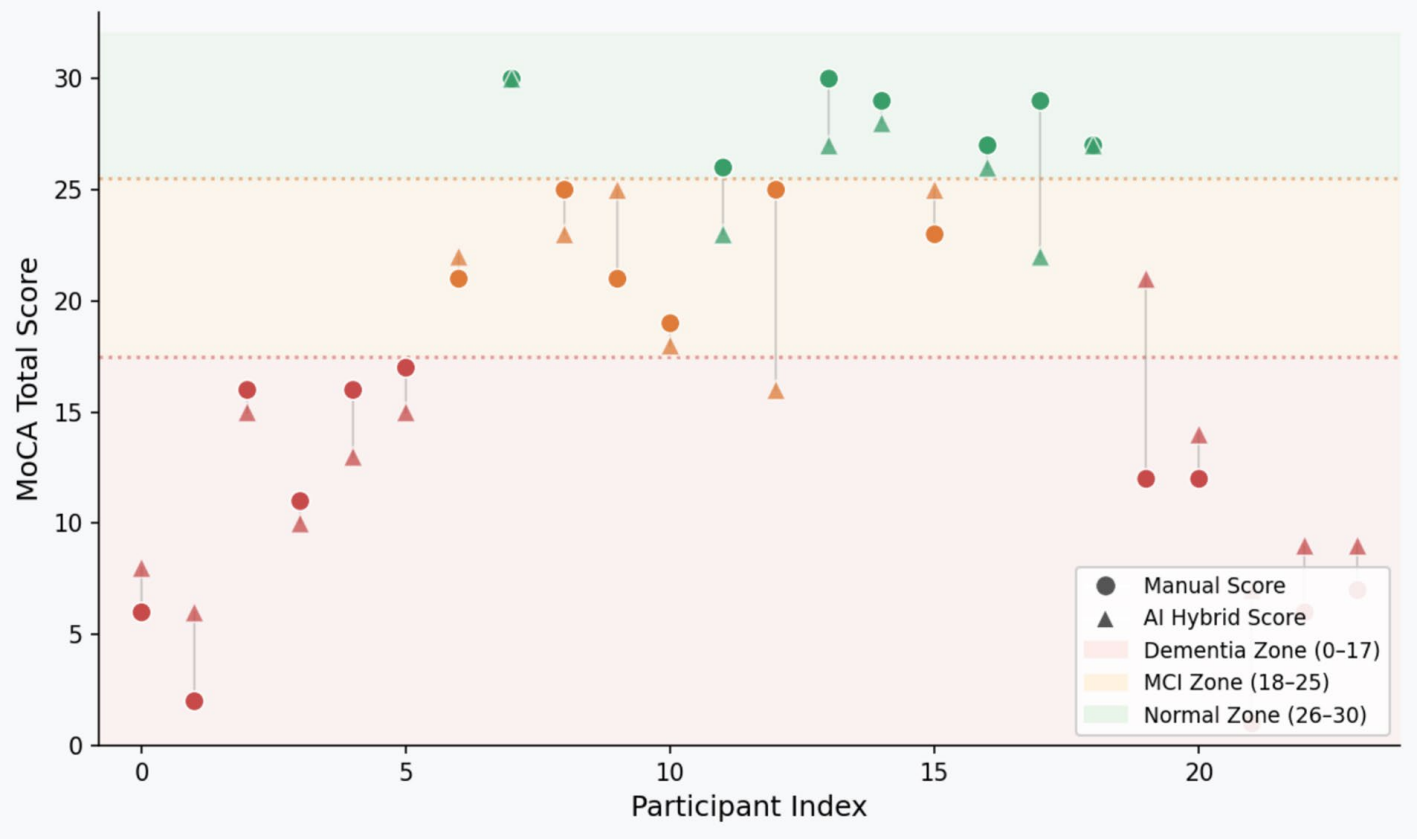
Manual vs. AI hybrid total MoCA score distribution across all 24 participants. Circles represent manual scores; triangles represent AI hybrid scores. Gray lines connect paired scores for each participant. Background shading indicates MoCA classification zones (red: Dementia, orange: MCI, green: Normal).

Figure 7 shows the distribution of manual and AI hybrid total scores across all 24 participants. Diagnostic boundary zones (Dementia: 0–17, MCI: 18–25, Normal: 26–30) are highlighted. Score discrepancies are visible only at boundary regions, reflecting the inherent difficulty of classifying borderline cases that differ between manual and AI scoring by only a few points.

### 5.7 Stability and repeatability

To assess output stability, the hybrid pipeline was executed five independent times on the same dataset. Identical final scores and diagnostic classifications were obtained across all runs, demonstrating complete operational stability despite the inclusion of constrained LLM components within the framework.

## 6 Discussion

This preliminary feasibility study evaluated a hybrid AI-assisted MoCA scoring framework against standardized manual MoCA assessment in Arabic-speaking participants. Given the pilot nature of this study, the findings should be interpreted as initial feasibility evidence rather than definitive clinical validation. The system achieved 83.3% overall diagnostic agreement, 90.9% sensitivity for Dementia, 83.3% for MCI, and 71.4% for Normal cognition, with Cohen’s *κ* = 0.74 (substantial agreement). Adjusted for technically compromised recordings, performance improved to 90.9% accuracy and *κ* = 0.86 (almost perfect agreement). Importantly, no severe cross-category misclassification (Dementia ↔ Normal) occurred.

One of the most clinically relevant findings is the complete absence of severe misclassification errors. No participant diagnosed manually with Dementia was classified as Normal by the AI system, and vice versa. All discrepancies occurred only between adjacent categories (MCI↔Dementia or MCI↔Normal), suggesting conservative diagnostic behavior, low false-reassurance risk, and alignment with clinical screening priorities. In cognitive screening contexts, false reassurance (classifying a individuals with dementia as normal) represents a far more critical risk than conservative over-referral. The absence of extreme diagnostic inversion strongly supports the safety profile of the hybrid architecture.

Moreover, the proposed system separates AI-assisted evaluation of 23 MoCA points from deterministic rule-based scoring of orientation and Letter-A tasks, preserving clinical transparency and traceability of domain-level scores. The system retains standard clinical cut-offs, ensuring direct comparability with manual assessment. This design aligns with growing recommendations in explainable AI for healthcare, emphasizing structured reasoning over black-box probabilistic outputs (Tjoa and Guan, 2020).

It is also worth noting that two misclassified Normal cases were associated with degraded audio recordings. Error propagation followed the pipeline of low audio quality, reduced ASR confidence, degraded linguistic feature extraction, lower language domain scores, and borderline classification shift. When excluding these cases, performance improved markedly, suggesting that performance degradation was primarily input-quality driven rather than a failure of the hybrid reasoning framework. These findings highlight the importance of standardized audio acquisition protocols in AI-assisted cognitive screening systems.

Previous AI-assisted cognitive screening studies have predominantly employed data-driven machine learning and deep learning pipelines, including end-to-end neural classifiers and probabilistic prediction models (Li et al., 2022). While these approaches demonstrated promising diagnostic performance, concerns remain regarding limited transparency and the persistent black-box characteristics of many AI systems in clinical contexts (Tjoa and Guan, 2020). The present study addresses these gaps by preserving official MoCA domain scoring logic while incorporating structured AI-assisted multimodal analysis within a hybrid explainable framework.

To our knowledge, this is also the first study to validate an AI-assisted MoCA scoring system specifically for Arabic-speaking populations using real-world clinical data, addressing a critical gap in the literature on culturally adapted cognitive screening tools (Kabalan et al., 2025; Taiebine, 2024).

### 6.1 Limitations and future directions

Several limitations must be acknowledged. First, the sample size (*n* = 24) is relatively small but it provides performance estimates for powering a full-scale validation trial. A prospective multi-center validation study with a target enrollment of at least 150–200 participants, stratified across cognitive categories, Arabic dialect regions, educational backgrounds, and clinical settings (tertiary hospitals, primary care clinics, and community-based rehabilitation centers), is planned as the next step. Second, the exploratory sociodemographic correlation analyses reported in Section 2.2 (education effect: *r* = 0.31, *p* = 0.14; no significant gender difference) are underpowered in this pilot cohort; adequately powered subgroup analyses examining education, gender, age, and dialect as moderators of AI-manual agreement are a primary objective of the planned follow-up study. Third, convenience sampling from a single elderly care facility may introduce selection bias. Fourth, two cases demonstrated sensitivity to degraded audio input, underscoring the need for integrated audio quality control mechanisms. Finally, while the Qwen2.5-7B model was applied under structured prompting constraints, non-determinism in LLM inference, though controlled via deterministic decoding settings (see Section 3.3.3), represents a potential source of variability for clinical deployment at scale.

Future research should validate the framework in larger multi-site cohorts across diverse Arabic dialects and educational backgrounds, integrate automatic audio quality detection with adaptive thresholding, investigate longitudinal monitoring capability for tracking cognitive trajectories, explore integration with primary care electronic health record systems, conduct prospective studies comparing the hybrid AI framework against specialized clinical assessment as a first-line screening tool, develop systematic ASR benchmarking against manually transcribed Palestinian and broader Levantine dialect speech corpora, incorporate quantitative neuropsychological linguistic features (type-token ratio, pause frequency, syntactic complexity) into the LLM-mediated scoring pipeline, and replace hard-coded geometric thresholds in the visuospatial modules with learned feature extraction layers to improve robustness in open-environment deployment.

## 7 Conclusion

This preliminary feasibility study demonstrates that a hybrid AI-assisted MoCA scoring system can achieve substantial agreement with manual clinical classification (83.3% accuracy; Cohen’s *κ* = 0.74) while preserving interpretability and diagnostic safety. These results should be interpreted as initial proof-of-concept evidence rather than definitive clinical equivalence, and replication in larger, adequately powered multi-site cohorts is required before broader conclusions can be drawn. The absence of severe cross-category misclassification and the improvement observed under controlled input conditions (*κ* = 0.86) support the feasibility of structured AI-assisted cognitive screening in real-world environments.

The proposed framework represents a clinically aligned, explainable, and culturally adaptable approach to AI-assisted neuropsychological assessment. By combining deterministic scoring logic with constrained LLM reasoning and robust safety overrides, the system bridges the gap between clinical neuropsychology, digital biomarker research, and explainable AI implementation, particularly for Arabic-speaking populations in low-resource settings.

## Data Availability

The datasets presented in this study can be found in online repositories. The names of the repository/repositories and accession number(s) can be found below: Dataset: https://github.com/yarajehadrabaya/arabic-moca-ai-dataset, and Code: https://github.com/yarajehadrabaya/moca-spaces.
